# Development of affordable 3D food printer with an exchangeable syringe-pump mechanism

**DOI:** 10.1016/j.ohx.2023.e00430

**Published:** 2023-05-18

**Authors:** Evren Demircan, Beraat Özçelik

**Affiliations:** a Istanbul Technical University, Department of Food Engineering, Turkey; b Bioactive Research & Innovation Food Manufac. Indust. Trade Ltd., Turkey

**Keywords:** 3D food printing, Additive manufacturing, Food extrusion mechanism

## Abstract

The technique of additive manufacturing has increasing popularity in food research area as well as other scientific fields. However, 3D food printers are expensive options compared to 3D polymer printers. Scientists, that require laboratory scale production capacities, resemble the syringe-pump systems that available in open source and free hardware designs. Present study aimed to develop an exchangeable syringe-pump mechanism (SPM) to demonstrate transformation of conventional 3D printer from polymer to food extrusion. The SPM can print a variety of materials, including miscellaneous foods, pastes, hydrogels and even biopolymers. The complete mechanism relies mostly on 3D printed parts and costs approximately 72$. Therefore, it allows users to obtain a 3D food printer inexpensively and does not require large amounts of technical labor. The SPM uses big volume (60 ml) luer lock syringe and blunt tip needles for greater versatility and user-friendliness. It could also be extended with cooling mechanism, so that the proposed system gains unique attribute among its counterparts. Finally, a standard polymer-printing 3D-Printer was converted into a laboratory-scale food printer, and edible ink was successfully printed in the desired shape.

## Graphical abstract

**Figure:**
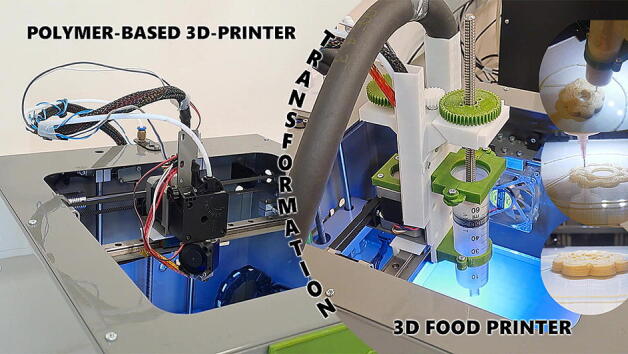

Specifications table.

**Table:** 

Hardware name	Syringe-pump mechanism (SPM)
Subject area	• Environmental, planetary and agricultural sciences • Educational tools and open source alternatives to existing infrastructure
Hardware type	• Mechanical engineering and materials science • 3D printer modification
Closest commercial analog	No commercial analog is available.
Open source license	CC-BY-SA 4.0
Cost of hardware	≈22$ (with cooling option it costs ≈72$)
Source file repository	https://doi.org/10.17632/5rdt5wmbv5.1

## Hardware in context

The technique of additive manufacturing (also known as 3D printing) offers high flexibility to designers and manufacturers in the fields of mechanical engineering, aeronautics, design science, biomedical engineering, pharmaceutical sector, biotechnology, and food science [1]. Additive manufacturing processes are generally classified in seven groups. These are binder jetting (BJT), directed energy deposition (DED), material extrusion (MEX), material jetting (MJT), powder bed fusion (PBF), sheet lamination (SHL) and vat photo polymerization (VPP) techniques. Material extrusion is a group of processes in which a thermo-plastic material (filament) is selectively applied layer by layer by being extruded through a nozzle that is also known as fused deposition modelling (FDM) [2]. Additive manufacturing or 3D printing has emerged as one of the most popular molding techniques in the food industry in recent years [3]. In food industry leading factors are health concerns (production of functional foods for specific target populations, such as elderly individuals who have trouble of chewing) and customer preferences (quest for novelty in sensory characteristics and dietary customization) [4]. To achieve these goals, specific type of printers that has capability of extrusion of food materials needed. Although there are different techniques that used in food industry, extrusion-based technologies are currently the most widely used technique. Over the years, three-dimensional printing has developed into a user-friendly and cost-effective craft. But it is still a very expensive process to implement these techniques in the field of food and biotechnological research and development in laboratories [5]. Numerous 3D printers for foods are commercially available right now. Prices cover a wide spectrum and some models are available approximately between 1000$ to 8500$ [6]. Extrusion based (fused deposition modelling, FDM type) models generally use syringe-pump systems. Scientists, that require laboratory scale production capacities, resemble the syringe-pump systems and can benefit from the availability of open source and free hardware designs which can be duplicated with inexpensive regular 3D printers [7]. In this context, some of syringe-pump based components/hardware assembly were presented in the literature [8], [9], [10], [11], [12], [13].

Aim of this study is to develop a syringe-pump mechanism that can be used in desktop 3D printers and that has capability of printing different food materials, pastes, hydrogels and even biopolymers and other shear thinning and elastic modulus fluids. The authors intended to add a cheap and simple syringe-pump system to the literature, open a gate to cost effective research and demonstrate transformation of 3D printer from polymer to food extrusion. With help of this mechanism, regular polymer based FDM type 3D printers could be converted to 3D food printers. Also with cooling mechanism attached, proposed system gains unique attribute in the field of food printers.

## Hardware description

This article describes development of a syringe pump mechanism (SPM) that converts extrusion-based FDM desktop 3D printer that is capable of extruding 1.75 mm thermo-plastic filaments, to a 3D food printer. At present, there are open source initiatives and companies offering cost-effective modifications to syringe pump extruders that can be used with inexpensive 3D printers. The PrintrBot Food & Paste Extruder is an example of such an option that is low-cost. However, it was specifically designed for the PrintrBot Simple Metal and requires rewiring during installation, which makes it difficult to adapt to other systems. The Replistruder by TJ Hinton is another syringe-based extruder that is custom-designed and open source, and has been utilized to demonstrate the printing of mechanically robust, biomimetic structures with high accuracy. However, it also has volume limitations and can only accommodate up to a 10 ml syringe as its ink reservoir. Although some open source designs, like the “Universal Paste Extruder” by RichRap, have been made available online for retrofitting 3D thermoplastic printers, these designs are often constrained by small build volumes or the inability to retract during printing [8].

The SPM was developed to present cheaper option of food printing 3D devices in scientific researches. As it compared with commercial 3D food printers, its cost is more affordable [6]. The main mechanism costs approximately 22$. If cooling option attached, the cost increases to 72$. For this study, the authors obtained a secondhand desktop 3D printer with less than 200$ so that total cost is not more than 300$. If a brand-new 3D printer is purchased, price range is between 200 and 500$ for a suitable printer [14]. At this point cost of total device is not more than 600$ which is nearly half of the cheapest option of 3D food printers [6]. Also the cost of main mechanism without cooling apparatus is nearly half of the open source syringe mechanisms with similar function [8], [10].

Researchers needing to 3D print food materials, pastes or similar fluid materials can benefit from the SPM. The mechanism is made up of two major parts: main component and an optional cooling apparatus. The main functional component of SPM consists of 23 parts in which 16 parts are 3D printable that makes it affordable and versatile. Since 3D printer materials are abundant, remaining parts are easily found items that include one trapezoidal lead screw and two brass nuts, luer-lock [15] syringe and three bearings. Auxiliary items are standard hardware such as M3/M5 bolts and nuts, and luer-lock needles. SPM can also be extended with attachment of cooling mechanism that comprised of aluminum cooling block, polyurethane tubing, tubing insulator, four 3D printed parts and recycled aluminum heatsink and fan from an old pc. Hence, there is the possibility of using waste material, the proposed hardware helps to recover unused components from old devices.

**Fig. 1:**
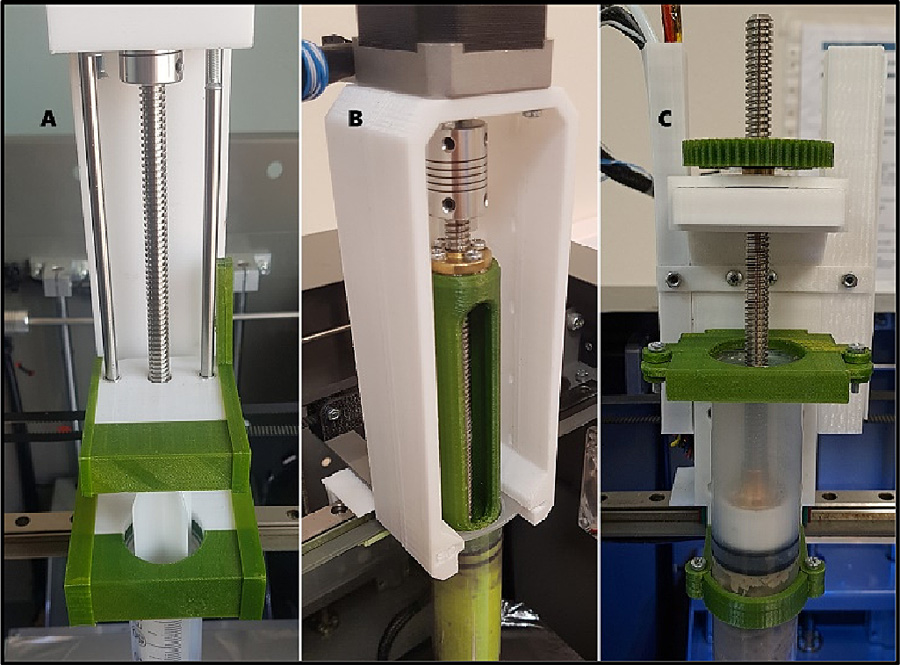
Mechanisms that built previously by authors: A was adapted from [17], B was from [18] and C is the current design.

The SPM relies on a guide carriage that mounted on a linear rail, which exists in some of regular 3D printers. It is a direct-drive system like some of its counterparts [13], [16]. The authors studied on two other different designs (unpublished work) that available in common 3D printing forums ([17], [18]) and adapted them to their desktop printer (Fig. 1). In these preliminary experiments, it is realized that total weight force of the mechanism, step motor and filled syringe was outside of linear rails that causes unstable pressure on the carriage and negatively effects movement which results in misplaced and uneven layers. The present study overcomes this problem by moving heavy piece, the step motor onto the carriage. Also current mechanism is smaller than these designs and occupies less space. Even difference is 1–2 cm on XY axis; big difference is on Z-axis, which is more than 7 cm that offers more volume of printing area.

A typical user can easily disassemble the mechanism (SPM), and the polymer extruder of the conventional 3D printer can be replaced back. Like as other open source 3-D printers, this gives opportunity of using same device for food materials and for thermoplastic filaments [9], [13], [12].

**Fig. 2:**
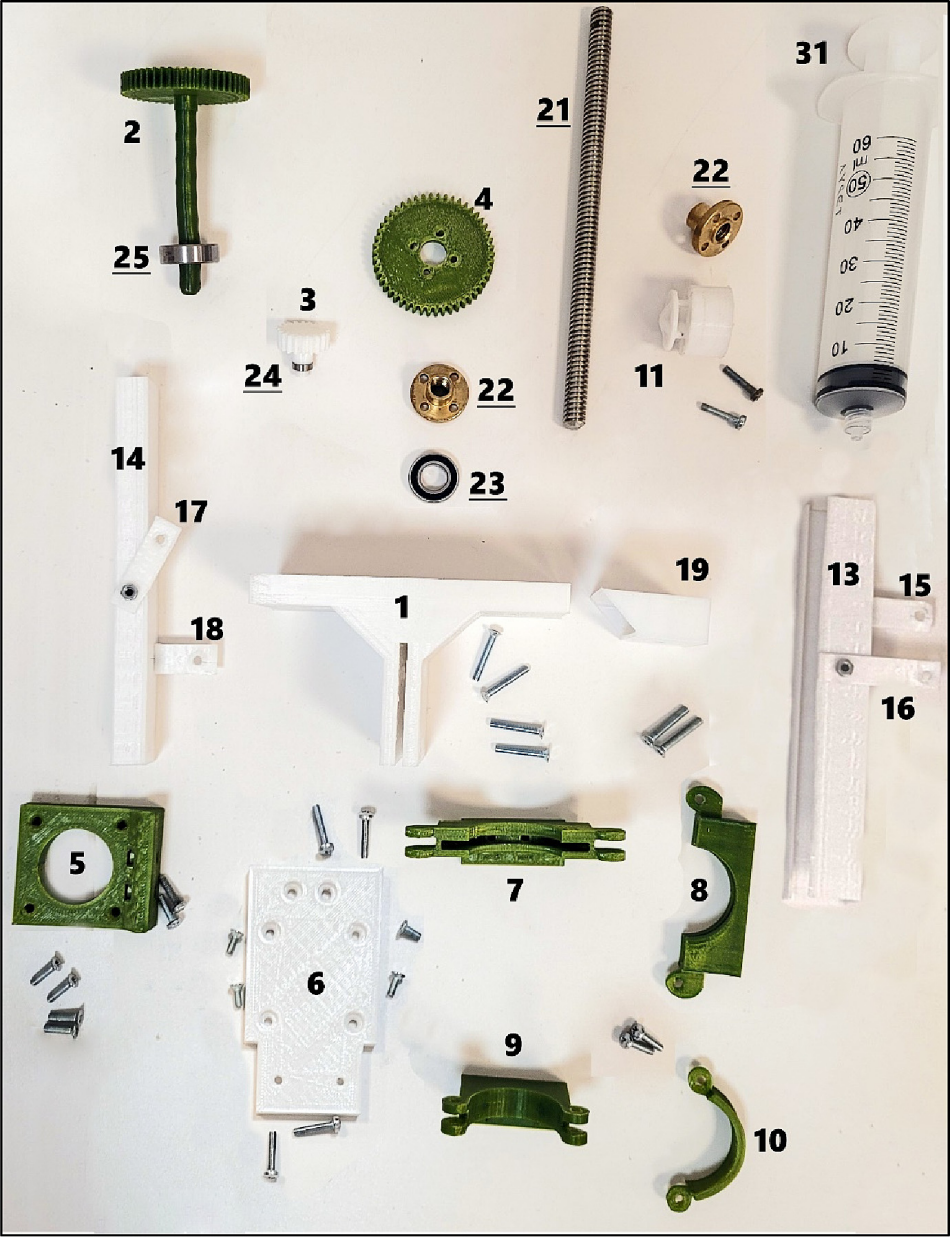
3D-printed parts (1–19), bearings (23–25), trapezoidal screw (21), nuts (22) and syringe (31) of SPM. Each number corresponds short name of 3D-printed parts (See “*Design files summary*” table). Underlined numbers are for non-printed parts (metal parts) which mentioned in “*Bill of materials summary*” table.

Currently SPM is compatible with 60 ml standard syringe that is widely available. However, it can be adapted to smaller or bigger volumes only by modifying syringe holder and plunger parts (Fig. 2, parts 7–11). This attribute gives SPM an advantage over its counterparts. [8], [12]. Syringes with the luer-lock attribute can accommodate various standard needle sizes that offered in the range of dimensional variations between 0.1 and 1.52 mm. Furthermore, SPM can work without needles so that working range is 0.1 mm to 1.6 mm.

Despite the extrusion with SPM conducted at room temperature, the proposed cooling apparatus allows the cooled air to be carried to the tip of the syringe. It provides an advantage over equivalent syringe pump mechanisms in terms of directly reaching the tip of the syringe [8], [9], [11], [13], [12], [19]. With this attribute, the layer stability of prints might be improved for some food materials whose viscosity increases under cold conditions.

There are many different types of food or bioprinters on the literature and they generally operate at low speeds [20], [21]. Since the mechanism we proposed is mounted on a linear rail guide and its center of gravity is designed to be on the rail, it can operate at relatively higher speeds than other printers. In addition, many syringe mechanisms are generally developed to fit Ender or Prusa models but cannot be used with linear rail guide mechanisms [8], [10], [13], [12]. Our system, on the other hand, is designed to achieve smoother motion with linear rail slides. Moreover, the SPM distinguishes from its counterparts thanks to its modular design. Modular design allows a heating pad can be attached to the syringe body, and by changing some parts of SPM, different syringe size could be used.

In summary, the offered syringe pump hardware has potential benefits in terms of undermentioned subjects especially for the researchers of food science;

• Introduced mechanism is cheap, open source, lightweight, and produced to be simple to modify.
• The SPM is exchanged with the extruder block of 3D printers and it uses stepper motor to drive the syringe plunger and relies on linear rails. So that, the mechanism operates similarly to conventional FDM polymer printers, making users with desktop 3D printers able to use it.
• It has relatively bigger size; 60 ml volume capacity that is sufficient for studies in the area of food research laboratories and can extrude in diameter range of 0.6 mm to 1.6 mm which results better layer stabilities.
• Syringe-pump mechanism can be used in standard 3D food printing tasks with relatively high speeds (more than 50 mm/s).

## Design files summary

**Fig. 3:**
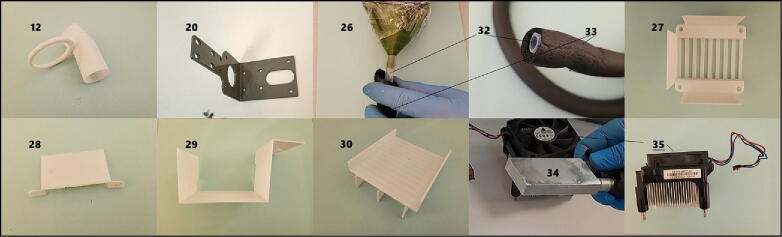
Carrier metal and parts for cooling apparatus: 3D-printed parts (12, 26–30), carrier metal (20), air tubing with insulation (32, 33), aluminum block (34) and cooling fan and heatsink (35). Each number corresponds to “*Design files summary*” and *Bill of materials summary*” tables.

Complete syringe pump mechanism relies mostly on 3D printed parts. Whole mechanism and all singular 3D-printing files were designed online in Tinkercad (Autodesk, Inc, USA). Related link of design file (CAD file) has been given in the table below (Table 1). Each part name has leading number which corresponds their short/part name at build instructions section and in Fig. 2 and Fig. 3. The photo (Fig. 2) has been taken in position that each part were close to its connecting part.

**Table 1:** Design files.

Design file name	File type	Open source license	Location of the file
Complete Syringe Pump Mechanism	CAD file	CC-BY-SA 3.0	https://www.tinkercad.com/things/l5lbcOVcHi1?sharecode=exSbfm9EREe6iNH42q_oMZHogvl0ZOBAJ2oJ1uTWg-w
01-MainBody.stl	STL file	CC-BY-SA 4.0	https://doi.org/10.17632/5rdt5wmbv5.1
02-Gear1.stl	STL file	CC-BY-SA 4.0	https://doi.org/10.17632/5rdt5wmbv5.1
03-Gear2.stl	STL file	CC-BY-SA 4.0	https://doi.org/10.17632/5rdt5wmbv5.1
04-Gear3.stl	STL file	CC-BY-SA 4.0	https://doi.org/10.17632/5rdt5wmbv5.1
05-MotorSupport.stl	STL file	CC-BY-SA 4.0	https://doi.org/10.17632/5rdt5wmbv5.1
06-MainBodyBottomExtention.stl	STL file	CC-BY-SA 4.0	https://doi.org/10.17632/5rdt5wmbv5.1
07-SyringeHolderTopMain.stl	STL file	CC-BY-SA 4.0	https://doi.org/10.17632/5rdt5wmbv5.1
08-SyringeHolderTopLocker.stl	STL file	CC-BY-SA 4.0	https://doi.org/10.17632/5rdt5wmbv5.1
09-SyringeHolderBottomMain.stl	STL file	CC-BY-SA 4.0	https://doi.org/10.17632/5rdt5wmbv5.1
10-SyringeHolderBottomLocker.stl	STL file	CC-BY-SA 4.0	https://doi.org/10.17632/5rdt5wmbv5.1
11-PistonHolder.stl	STL file	CC-BY-SA 4.0	https://doi.org/10.17632/5rdt5wmbv5.1
12-CoolerPipeHolderBottom.stl	STL file	CC-BY-SA 4.0	https://doi.org/10.17632/5rdt5wmbv5.1
13-CoolerPipeHolderDuct.stl	STL file	CC-BY-SA 4.0	https://doi.org/10.17632/5rdt5wmbv5.1
14-CableHolderDuct.stl	STL file	CC-BY-SA 4.0	https://doi.org/10.17632/5rdt5wmbv5.1
15-PipeDuctTopSupport.stl	STL file	CC-BY-SA 4.0	https://doi.org/10.17632/5rdt5wmbv5.1
16-PipeDuctBotomSupport.stl	STL file	CC-BY-SA 4.0	https://doi.org/10.17632/5rdt5wmbv5.1
17-CableDuctTopSupport.stl	STL file	CC-BY-SA 4.0	https://doi.org/10.17632/5rdt5wmbv5.1
18-CableDuctBotomSupport.stl	STL file	CC-BY-SA 4.0	https://doi.org/10.17632/5rdt5wmbv5.1
19-BearingKeeper.stl	STL file	CC-BY-SA 4.0	https://doi.org/10.17632/5rdt5wmbv5.1
20-CarrierMetal.stl	STL file	CC-BY-SA 4.0	https://doi.org/10.17632/5rdt5wmbv5.1
26-Funnel_forCooler.stl	STL file	CC-BY-SA 4.0	https://doi.org/10.17632/5rdt5wmbv5.1
27-BaseForAluminiumBlock_forCooler.stl	STL file	CC-BY-SA 4.0	https://doi.org/10.17632/5rdt5wmbv5.1
28-BaseCoverForAluminiumBlock_forCooler.stl	STL file	CC-BY-SA 4.0	https://doi.org/10.17632/5rdt5wmbv5.1
29-CoolerHolder.stl	STL file	CC-BY-SA 4.0	https://doi.org/10.17632/5rdt5wmbv5.1
30-CoolerHolderBase.stl	STL file	CC-BY-SA 4.0	https://doi.org/10.17632/5rdt5wmbv5.1

**01-MainBody *(***Fig. 2***no.1):*** This part is main body of mechanism. It is the part that connects gears and holds other parts.

**02-Gear1 *(***Fig. 2***no.2)*:** Gear with leg. This gear transmits the movement of step motor.

**03-Gear2 *(***Fig. 2***no.3)*:** It transmits the movement between gear 1 and gear 3.

**04-Gear3 *(***Fig. 2***no.4)*:** It transmits the movement to lead screw that attached to the plunger in the syringe.

**05-MotorSupport *(***Fig. 2***no.5)*:** This part fixes step motor’s position and attaches it to the body of mechanism.

**06-MainBodyBottomExtention *(***Fig. 2***no.6)*:** It is the extension of main body and it holds syringe’s bottom part.

**07-SyringeHolderTopMain *(***Fig. 2***no.7)*:** Holds the syringe. Syringe flange is inserted in this part.

**08-SyringeHolderTopLocker *(***Fig. 2***no.8)*:** Holds the syringe. Syringe flange gets in this part. Locking mechanism for part 7.

**09-SyringeHolderBottomMain *(***Fig. 2***no.9)*:** Syringe collar that constrains the syringe.

**10-SyringeHolderBottomLocker *(***Fig. 2***no.10)*:** Syringe collar that constrains the syringe. Locking mechanism for part 9.

**11-PistonHolder *(***Fig. 2***no.11)*:** A short plunger that attached to the trapezoid screw.

**12-CoolerPipeHolderBottom *(***Fig. 3***no.12)*:** This pipe holder part is used to keep end of air tubing closer to the syringe tip. It has ring attached that fixes position.

**13-CoolerPipeHolderDuct *(***Fig. 2***no.13)*:** Semi-open duct part is used to arrange air tubing, to prevent entanglement. During implementation of tubing, it can be bended or stretched out. It is better that this part printed with high tensile strength filaments like PETG.

**14-CableHolderDuct *(***Fig. 2***no.14)*:** Cable holder duct is also used for arrangement and preventing entanglement of cables. And same like part 13, it is advised that this part printed with PETG.

**15-PipeDuctTopSupport *(***Fig. 2***no.15)*:** Connects duct (part 13) to the main body.

**16-PipeDuctBotomSupport *(***Fig. 2***no.16)*:** Connects duct (part 13) from back side to the main body.

**17-CableDuctTopSupport *(***Fig. 2***no.17)*:** Connects cable duct (part 14) to the main body.

**18-CableDuctBotomSupport *(***Fig. 2***no.18)*:** Connects duct (part 14) from back side to the main body.

**19-BearingKeeper *(***Fig. 2***no.19)*:** Prevents the bearing (part 23) from slipping because of backpressure force.

**20-CarrierMetal *(***Fig. 3***no.20)*:** This component can be either metal or 3D-printed. If 3D-printed, 100% infill advised. Carrier metal is important part of mechanism. It is used to fixate mechanism on to the linear rails of 3D-printer.

**26-Funnel_forCooler *(***Fig. 3***no.26)*:** Helps to redirect air from cooling fan to the polyurethane pipe.

**27-BaseForAluminiumBlock_forCooler *(***Fig. 3***no.27)*:** Place for aluminum block in which cooling water flows. This part keeps aluminum block and fan together. Prevents air from outside of cooling block.

**28-BaseCoverForAluminiumBlock_forCooler *(***Fig. 3***no.28)*:** Coverage part for the bared side of part 27.

**29-CoolerHolder *(***Fig. 3***no.29)*:** Keeps cooling mechanism holded on the printer.

**30-CoolerHolderBase *(***Fig. 3***no.30)*:** Bottom section of part 29. With its triangle bottom, it supports cooler apparatus to lean on the printer.

## Bill of materials summary

Since most of the materials are 3d printed, in the BOM list (Table 2) the quantities of each filament used are given in a single line. The amount of filament consumed and price information for each part are also given in separate list (Table 3). Although filaments are sold in certain grams, and some parts like bolts, nuts are sold with minimum amount, the prices stated here are given for producing single SPM mechanism. Price of cooling fan and heatsink (part 35) can be deducted from total cost because it can be obtained by recycling from an old pc. Moreover, prices for shipment and required hand tools are disregarded. If cooling part is used, there should be recirculating water chiller. Since proposed mechanism will be used in research laboratories, it is assumed that this apparatus exists in regular laboratories and price of chiller is also neglected.

**Table 2:** Bill of materials (BOM).

Designator	Component	Number	Cost per unit -currency	Total cost – currency	Source of materials	Material type
Parts for Body						
Parts (1,3,6,11–20 and 26–30)	PETG Filament	292 g	20.00 $/kg	5.86 $	Amazon	Polymer
Parts (2,4,5,7–10)	PLA Filament	56 g	20.00 $/kg	1.13 $	Amazon	Polymer
Part 21 and 22	T8 Trapezoidal Lead Screw + T8 Nut, Pitch 2 mm Screw Lead 8 mm	1	7.62 $	7.62 $	Amazon	Metal
Part 22	T8 Nut	1	2.50 $	2.50 $	Amazon	Metal
Part 23	6800RS Ball Bearing 10x19x5mm	1	1.20 $	1.20 $	Amazon	Metal
Part 24	MR85-2RS Ball Bearing 5x8x2.5 mm	1	1.00 $	1.00 $	Amazon	Metal
Part 25	608 ZZ Double Shielded,8x22x7 Ball Bearing	1	0.64 $	0.64 $	Amazon	Metal
Part 31	60 ml Luer-Lock Syringe	1	1.00 $	1.00 $	Amazon	Polymer

Parts for Cooler						
Part 32	Polyurethane Tubing, 3/8″ OD, 8/16″ ID	1.5 *m*	3.00 $	4.50 $	Amazon	Polymer
Part 33	Foam Tubing for Pipe Insulation	1.5 *m*	15.00 $	22.50 $	Amazon	Polymer
Part 34	Aluminium Block (40×80×12 mm)	1	10.00 $	10.00 $	Amazon	Metal
Part 35	Cooling Fan and heatsink	1	13.00 $	13.00 $ *	Amazon or obtained by recycling.	Polymer/Metal

Bolts and Nuts						
	M3x6mm bolts	4	0.01 $	0.04 $	Local hardware store	Metal
	M3 × 6 mm bolts with hex socket head	4	0.01 $	0.04 $	Local hardware store	Metal
	M3x8mm bolts	4	0.01 $	0.04 $	Local hardware store	Metal
	M3x10mm bolts	10	0.01 $	0.10 $	Local hardware store	Metal
	M3x12mm bolts	6	0.01 $	0.06 $	Local hardware store	Metal
	M3x15mm bolts	2	0.01 $	0.02 $	Local hardware store	Metal
	M3x20mm bolts	2	0.02 $	0.04 $	Local hardware store	Metal
	M3 Hex Nuts	20	0.01 $	0.20 $	Local hardware store	Metal
	M4x10mm bolts	4	0.02 $	0.08 $	Local hardware store	Metal
	M4 Hex Nuts	4	0.02 $	0.08 $	Local hardware store	Metal
	M5x30mm bolt	1	0.02 $	0.02 $	Local hardware store	Metal
	M5 Hex Nuts	1	0.02 $	0.02 $	Local hardware store	Metal

**Table 3:** List of 3D printed parts, required filament type, length and cost of each part.

Part Name	Weight (g)	Length (m)	Cost ($)	Type of Filament
01-MainBody.stl	57	18.50	1.13 $	PETG
02-Gear1.stl	11	3.65	0.22 $	PLA
03-Gear2.stl	2	0.57	0.03 $	PETG
04-Gear3.stl	8	2.74	0.16 $	PLA
05-MotorSupport.stl	11	3.64	0.22 $	PLA
06-MainBodyBottomExtention.stl	16	5.26	0.32 $	PETG
07-SyringeHolderTopMain.stl	11	3.54	0.21 $	PLA
08-SyringeHolderTopLocker.stl	7	2.46	0.15 $	PLA
09-SyringeHolderBottomMain.stl	6	2.15	0.13 $	PLA
10-SyringeHolderBottomLocker.stl	2	0.75	0.04 $	PLA
11-PistonHolder.stl	8	2.65	0.16 $	PETG
12-CoolerPipeHolderBottom.stl	6	2.12	0.13 $	PETG
13-CoolerPipeHolderDuct.stl	15	4.86	0.30 $	PETG
14-CableHolderDuct.stl	12	4.09	0.25 $	PETG
15-PipeDuctTopSupport.stl	1	0.22	0.01 $	PETG
16-PipeDuctBotomSupport.stl	1	0.17	0.01 $	PETG
17-CableDuctTopSupport.stl	1	0.17	0.01 $	PETG
18-CableDuctBotomSupport.stl	0	0.16	0.01 $	PETG
19-BearingKeeper.stl	9	3.10	0.19 $	PETG
20-CarrierMetal.stl	19	6.28	0.40 $	PETG
26-Funnel_forCooler.stl	37	12.08	0.74 $	PETG
27-BaseForAluminiumBlock_forCooler.stl	36	11.66	0.71 $	PETG
28-BaseCoverForAluminiumBlock_forCooler.stl	3	1.08	0.07 $	PETG
29-CoolerHolder.stl	43	13.95	0.85 $	PETG
30-CoolerHolderBase.stl	26	8.67	0.53 $	PETG
*Total PETG Filament*	*292 g*	*96 m*	*5.86 $*	
*Total PLA + Filament*	*56 g*	*19 m*	*1.13 $*	
**TOTAL PRICE**			**6.99 $**	

All materials might be found online, at any business that specializes in 3D printers, or at any hardware store. Here a widespread online store link suggested for equivalent materials.

## Build instructions

**Fig. 4:**
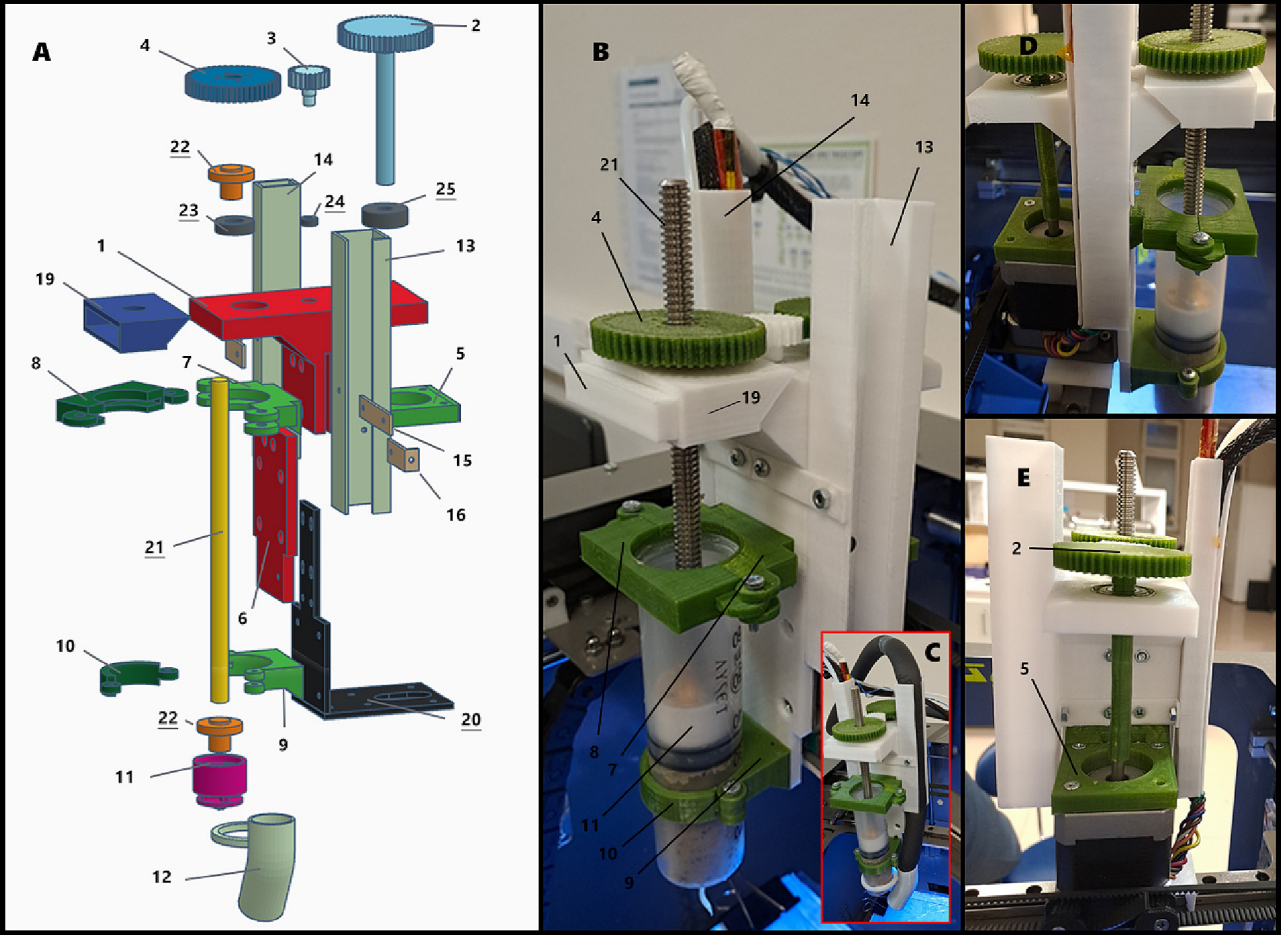
**A.** Orientation shown on the CAD drawing of SPM. Each number is mentioned in “*Design files summary*”, “*Bill of materials summary*” tables and build instructions section. **B.** Constructed SPM mechanism (left-view). **C.** Mechanism with cooling apparatus attached. **D.** Right view. **E.** Rear view.

A Rigid3D Zero 2 (Rigid3D Corp., Türkiye) printer [22] with Titan Extruder [23] used in this experiment. A screwdriver, M3 and M1.4 Allen wrenches, M8 wrench and tweezers are required tools for the assembly. If cooling part is used, there also should be recirculating water chiller. Part numbers associated in this section, can be seen in Fig. 2, Fig. 3, Fig. 4, which are same in design files table (Table 1) and BOM table (Table 2). Supplementary video is provided for comprehensive step-by-step building instructions as well. Furthermore, Fig. 4 is self-explanatory for orientation and position of items.

### Preliminary steps

**Step 1:** Print parts 1, 3, 6, 11–20 and 26–30 with PETG filament in 3D printer. Set the 3D-printing settings to: 25% infill, three layers at bottom and top, 0.2 mm layer height and 1.2 mm wall thickness.

**Step 2:** Change filament to PLA+ and print parts 2, 4, 5 and 7–10 with same settings (except temperature of extrusion, use recommended printing temperature from the filament supplier).

**Step 3:** Laser cut a metal to obtain part 20. Even part 20 is metal, it can be printed with PETG. If it is 3D-printed, 100% infill setting recommended for increasing the strength.

### Removing filament extruder

**Step 4:** Shut down desktop 3D printer and unplug electricity cable. Disassemble the extruder and hotend parts of 3D desktop printer with M3 and M1.4 Allen wrenches. Only the extruder's step motor and linear guide carriage on the rail should remain in place of the extruder. Cables of the extruder fan will be used for the cooling fan. In case of an unexpected temperature rise, cover the heating cartridge with an insulator, such as teflon tape.

Part 20 is the important item of SPM that mounts the mechanism to the linear guide carriage. In this hardware, holes at the bottom of part 20 are for MGN12H carriage and connection of GT2 timing belt holder. If different carriage was used part 20 should be adapted for that carriage.

### Preparing main components

**Step 5:** Mount part 20 to the carriage on the linear rails, with four pieces of M3 × 6 mm screws. Attach it to the GT2 timing belt holder with two of M3 × 12 mm screws.

#### Building the main body

**Step 6:** Insert part 2 into the 608ZZ bearing (part 25) and mount them into part 1.

**Step 7:** Insert 6800RS bearing (part 23) into part 1 and cover it with part 19. Slide part 19 until holes become concentric with bearing hole.

**Step 8:** Combine part 4 and part 22 and mount them onto the part 1 by inserting them into the part 19 hole. Orientation of part 22 should be long-side down. During joining part 4 and part 22, you may need four pieces of M3 × 10 bolts and nuts, but they are not necessary since printed part (4) is close-fit.

**Step 9:** Insert part 3 into the MR85-2RS bearing (part 24) and mount them into part 1 between part 2 and part 4 gears. Check for transmission, if they are connected.

**Step 10:** Insert two M3 nuts into the corresponding hex hole at the foot of part 1. Mount main body onto the part 20.

#### Preparing the step motor

**Step 11:** Insert two of M3 nuts into part 5. Mount part 5 onto the step motor with four pieces of M3 × 8 mm screws. Step motor is extruder’s motor that obtained at step 4. Motor’s cable position should be left-hand side, while positioning back of part 5 to yourself (see Fig. 4).

#### Preparing the main body bottom extension

**Step 12:** Part 6 has holes that matched with part 20 when placed under the foot of part 1. So that two holes at the bottom of part 6 is the place of part 9. Insert two of M3 nuts into part 9. Install part 9 onto the bottom side of part 6 with two pieces of M3 × 12 mm screws. Orientation of part 9 is that triangle section should be downside.

**Step 13:** Attach part 10 to the part 9 from holes with two screws (M3 × 8 mm and M3 × 20 mm). No need to turn the screws, they are needed to hold parts.

#### Preparing the cable holder duct

**Step 14:** Attach part 17 and part 18 to the part 14 with two M3 × 6 mm screws with hex socket head and nuts. See orientation and place of items in Fig. 2. Keep nuts outside. Use M1.4 Allen wrench, hold screw from the slit and insert nut.

#### Preparing the cooler tubing holder duct

**Step 15:** Attach part 15 and part 16 to the part 13 with two M3 × 6 mm screws with hex socket head and nuts. See orientation and place of items in Fig. 2, Fig. 4. Keep nuts outside. Use M1.4 Allen wrench, hold the screw from the slit and insert nut.

#### Preparing the plunger

**Step 16:** Insert two M3 nuts into the nut holder of part 11. Insert part 22 into the part 11 with two pieces of M3 × 12 mm screws. See orientation of part 22 in Fig. 4.

**Step 17:** Remove the original plunger from syringe (part 31). Get the elastic tip of plunger and mount it on the part 11.

**Step 18:** Screw trapezoidal T8 rod (part 21) into the part 22 that attached to the part 11. Turn it to the end.

**Step 19:** Insert the newly made plunger into syringe (part 31).

### Installing SPM

In this section, components that were prepared in “Preparing main components” section are assembled onto the part 20.

**Step 20:** Mount main body (Steps 6-10) on the top of the part 20. Long-legged gear should face to back of printer (see Fig. 4).

**Step 21:** Attach main body bottom extension (Steps 12-13) to the part 20 with four pieces of M3 × 10 mm screws and nuts.

**Step 22:** Put step motor component (Step 11) on the part 20, on the linear rail. Insert motor shaft into gear leg (part 2) and put component (Step 11) under the main body component (Steps 6-10). Use two of M3 × 15 mm screws to attach step motor component (Step 11), part 20 and extension component (Steps 12-13). Bolts should reach the nuts that are inside of part 5.

**Step 23:** Take the cable duct component (Step 14) and tuck cables and cable-like pieces into the duct by stretching the slit edge of the duct. Position the duct to the left-hand side of main body (Steps 6-10), and move it up so that part 17 comes to edge of slope where holes with hex nuts (at step 10) become concentric. Use an M3 × 15 mm screw and attach top of duct to that nut.

**Step 24:** Take the cooler tubing holder duct component (Step 15), position it on the right-hand side of main body (Steps 6-10) and move up like step 22, so that part 15 comes to edge of slope where holes with hex nuts (at step 10) become concentric. Use an M3 × 15 mm screw and attach top of duct to that nut. See orientation of duct in Fig. 4.

**Step 25:** Add two of M3 hex nuts into part 7. Attach part 7 to main body (Steps 6-10) with two of M3 × 15 mm screws. Insert screws from the backside by merging bottom hole of cable duct component (Step 14) and the tubing duct component (Step 15). Orientation of part 7 is that triangle section should be downside.

**Step 26:** Attach part 8 to the part 7 from holes with two screws (M3 × 8 mm and M3 × 20 mm).

**Step 27:** Remove the plunger from the syringe (Steps 16-19) until it reaches the top of the syringe. Then unscrew T8 rod (part 21). Remove long screws from syringe holder parts (from part 7–8 and part 9–10) and open them like a gate. Install syringe (part 31) into part 7 and lock it with part 8 by reattaching long screw. Do same for the parts 9 and 10.

**Step 28:** Screw the T8 rod (part 21) from the top of the gear (part 4) passing through it and getting inside of plunger (Steps 16-19). While screwing part 21 hold the plunger with your finger to let screw in. Turn the gears and check for plungers up-down movement.

## Building cooling apparatus

**Step 29:** Merge part 29 and part 30 by inserting legs of part 30 into corresponding slits on the part 29. Hang this component to the printer with M5 × 30 mm bolt and nut from the hole of part 29.

**Step 30:** Insert polyurethane tubing (part 32) inside of foam tubing for pipe insulation (part 33). Attach tubing to the funnel (part 26) by hand (Fig. 3).

**Step 31:** Attach aluminum block (part 34) to the inlet and outlet of a recirculating water chiller or similar equipment. Then put it into the part 27.

**Step 32:** Put part 28 onto the cooling fan and heatsink (part 35). Then mount part 27 that containing aluminum block (part 34) on it. The part 27 and part 28 should cover all sides of heatsink. Tighten with four of M4 nuts.

**Step 33:** Mount the part 26 with tubing (prepared at step 30) onto the cooling fan with four of M4 × 10 mm bolts (In supplemented video we used adhesive tape and extended the way of air with some of cardboard). Put the resulted component onto the hanged part (prepared at step 29).

**Step 34:** Insert opening end of tubing into the part 12 then move tubing within the duct (Step 15) and attach part 12 to the tip of syringe (part 31).

**Step 35:** Connect cables that were removed from extruder’s hotend fan (at step 4) to the pins of cooling fan.

## Operation instructions

Follow these instructions to properly operate the 3D Food Printer:

• Prepare desired shape in a CAD program. Get*.stl* file and obtain g-code file by slicing it in a slicer program.
• Add related g-codes in the beginning of file to disable heating of bed and extruder, homing the mechanism.
• Plug the electricity cable, switch on the food printer. If using cooler apparatus, switch on the chiller.
• Remove plunger rod (Fig. 4B no.21) from syringe by turning gears (Fig. 4B no.4), take syringe and fill it with food (or other paste like) material.
• Set the flow rate to 8% and extrusion temperature below 20°C.
• Set filament diameter and layer height corresponding with size of the needle.
• Insert the syringe into the holder (Fig. 4B no.7) and lock both of the bottom and upper clamps (Fig. 4B no.8 and 10) with bolts.
• Turn gears (Fig. 4B no.4) counter-wise until plunger (Fig. 4B no.11) gets in the syringe. Hold the rod (Fig. 4B no.21) with your fingers during turning gears to lead movement of rod. Remove waste that comes out from the tip of syringe.
• Start printing process on the device or from the computer.

## Safety precautions:

• Be careful not to pinch your fingers between gears during operation.
• Since extrusion temperature set to below the room temperature, be careful about heating cartridge of previous printer as it exposed to air.

## Possible drawbacks and solutions:

• 3D printers have a safety feature that prevents cold extrusion to avoid damaging extruder when there are issues with the hot end. In this case, add *M302 S0* code to the beginning of g-code file, which permits cold extrusion.
• The extruder motor could be inverted in the default configuration, thus during operation it can be seen that the T8 rod moving on the opposite direction. Add *M92 Z-400* command by providing negative step/mm values. Change step value (4 0 0) according to your printers setting.
• There may be issues with air flow rate of cooling apparatus. In this case, replace the fans with higher-powered ones.

## Limitations:

• The SPM is connected to a linear guide carriage in order to minimize vibrations during movement. In this case, the proposed system is not suitable for use in devices that do not have a linear rail carriage such as the common sling bed printers (Prusa [24] or Ender [25]). However, thanks to the modular structure of the proposed system, it can be adapted to this type of devices by changing/modifying only one part (Fig. 4A no.20) without changing the whole system. Either part of SPM could be modified or a linear rail upgrade could be done for the printers ([26], [27]).

## Validation and characterization

The printing assessment of SPM was conducted by a factorial design. A hollow cylindrical shape was printed in size of (40 mm × 40 mm × 20 mm) and the factors including nozzle height (NH, 0 mm-3.30 mm), printing speed (PS, 20-80 mm/s) and flow compensation (FC, 90-100%) were inspected using full factorial design in our another study [28]. As a result of experimental design, optimum parameters were selected for NH 0 mm, PS 50 mm/s and FC 100%. As the rheological behavior influences the extrusion, printability evaluation conducted with three different food paste differing in their viscosity [29]. To validate the working of SPM and observe possible printing capabilities of different nozzles, an oleogel mixture (OG) prepared to assess 0.6 mm diameter nozzle, a mushroom fortified semi-solid food mixture (MF) for dysphagia patients prepared to assess 1.2 mm nozzle and a meat analogue (MA) prepared to assess 1.6 mm nozzle [30], [31], [32]. A flower structure and hollow circle were drawn in Tinkercad (Autodesk, Inc.) and exported as a.stl file and sliced with Ultimaker Cura 5.0 [33], [34]. Printing of edible inks were conducted with settings in Table 4.

**Table 4:** Printing parameters of food inks.

Food Ink	OG	MF	MA
Nozzle diameter, mm	0.6	1.2	1.6
Line width, mm	0,6	1,2	1,6
Layer height, mm	0,6	1,2	1,6
Nozzle height, mm	0	0	0
Retraction, mm/s	10	0	15
Printing speed, mm/s	50	50	50
Flow compensation, %	100	100	100
Shape	Hollow Cylinder	Flower	Hollow Cylinder
Size (w × l × h), mm	40×40×3	47×47×6	40×40×20
Hollow size, mm	25	18	25
Number of layers	5	5	12

**Fig. 5:**
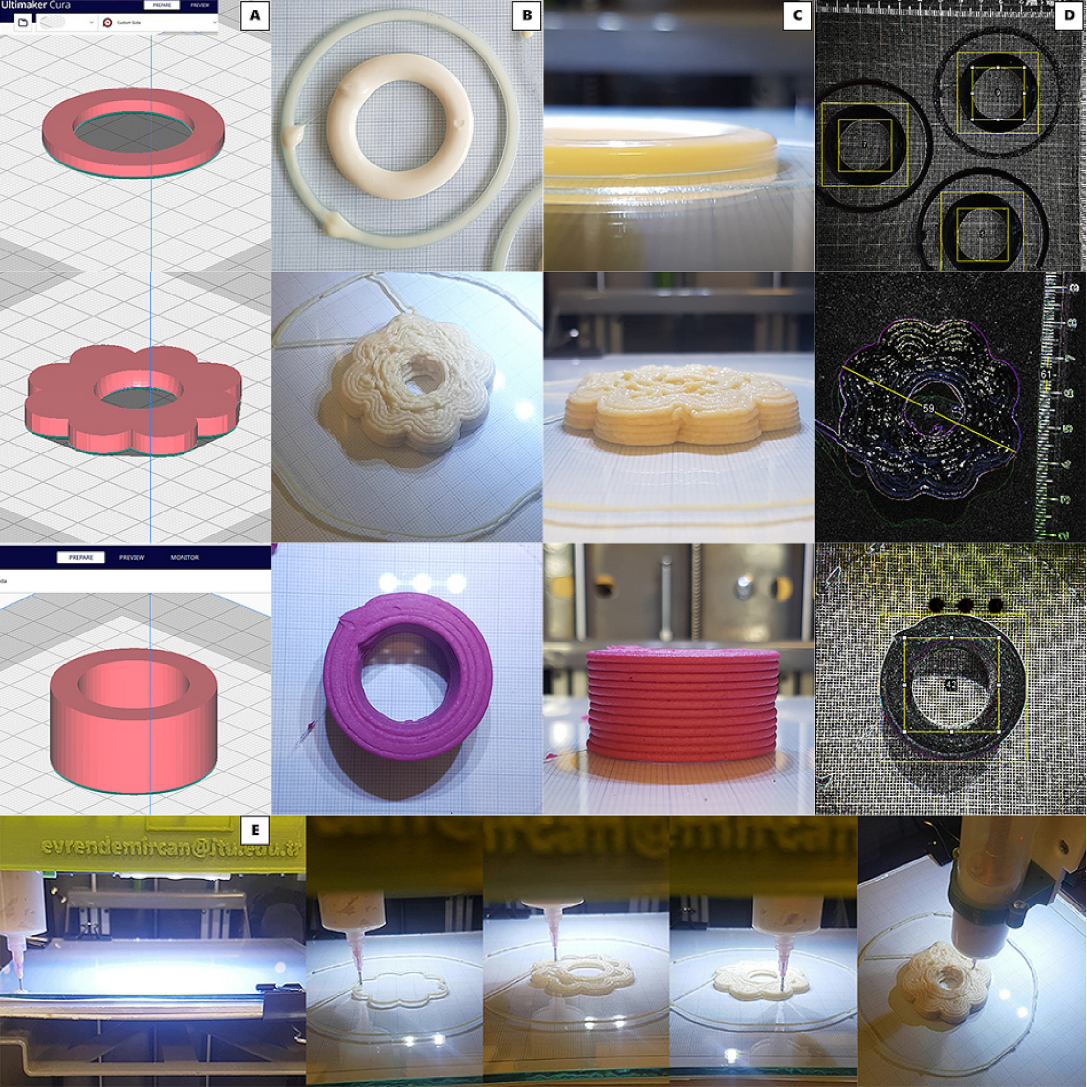
3D printing of three different food inks (OG, MF, MA and progress of MF; top-down respectively). **A)** stl files in Cura, **B)** Top views, **C)** Side views, the layers of printed food materials, **D)** Images from ImageJ, **E)** The SPM in-progress.

Resulting prints were analyzed with image processing programme, ImageJ, in order to accurately measure the perimeters and the gaps (Fig. 5) [35]. Each formulation was printed at least in triplicate and shape fidelity calculated with the equation below [36].

$$Shape\;fidelity=\frac{Measured\;dimension}{Theoretical\;dimension}\times 100$$

Hollow cylinders and flower shapes were successfully 3D-printed with three different food inks (Fig. 5). Shape fidelity values of diameters ranged between 99.3 and 100.7 %, heights ranged between 100.2 and 100.8% and accuracy of gap size ranged between 99.0 and 102.3 % (Table 5). Results showed that at high layer numbers, deviation of height accuracy increased, this could be related with rheological properties of food materials. However deviation of all dimensions were not higher than 3% which demonstrates that the performance of the SPM was satisfactory for the food materials. Flower shape were 3D-printed without retraction, on the other hand during printing of cylinders different retraction speeds were used. A filamentous structure could be observed between the skirt and the print body when no retraction was used however with relatively lower retraction speed OG sample accumulated and represented a ridge on the retraction point (Fig. 5.B).

**Table 5:** Accuracy assessment of printed food inks.

**Nozzle diameter, mm**	0.6	1.2	1.6
**Food Ink**	**OG**	**MF**	**MA**
**Shape fidelity, %**			
Width (diameter, y axis)	100.2±2.1	99.3±0.8	100.7±1.3
Length (diameter, x axis)	99.8±1.6	99.9±0.5	100.2±0.7
Height	100.5±0.6	100.2±2.1	100.8±1.5
Hollow size	99.7±0.9	99.0±1.3	102.3±0.9

In conclusion, an extrusion-based FDM desktop 3D-printer was modified by replacement of extruder part with our proposed mechanism, SPM. With the attachment of SPM, 3D-printer was converted to food printing capable laboratory scale 3D food printer and various shapes with different nozzle sizes and variable layers, were successfully printed. This research showed that the visual acceptance of paste-like foods can be increased by SPM-mounted 3D food printers, so that they can be a possible solution for malnutrition in patients. The SPM exposed that it could be reliable and inexpensive option for the laboratory size 3D food printers.

## CRediT authorship contribution statement

**Evren Demircan:** Conceptualization, Methodology, Software, Writing – original draft. **Beraat Özçelik:** Supervision, Writing – review & editing.

## Declaration of Competing Interest

The authors declare the following financial interests/personal relationships which may be considered as potential competing interests: Evren Demircan and Beraat Özçelik have patent application with collaboration of İTÜNOVA Teknoloji A.Ş.
