# Supplementary material for: Development of affordable 3D food printer with an exchangeable syringe-pump mechanism
Source: HardwareX. 2023 May 18;14:e00430. doi: 10.1016/j.ohx.2023.e00430 (PMC10225918; doi:10.1016/j.ohx.2023.e00430)

**Figure S1.** The 3D Printer on which the SPM mounted (front view).


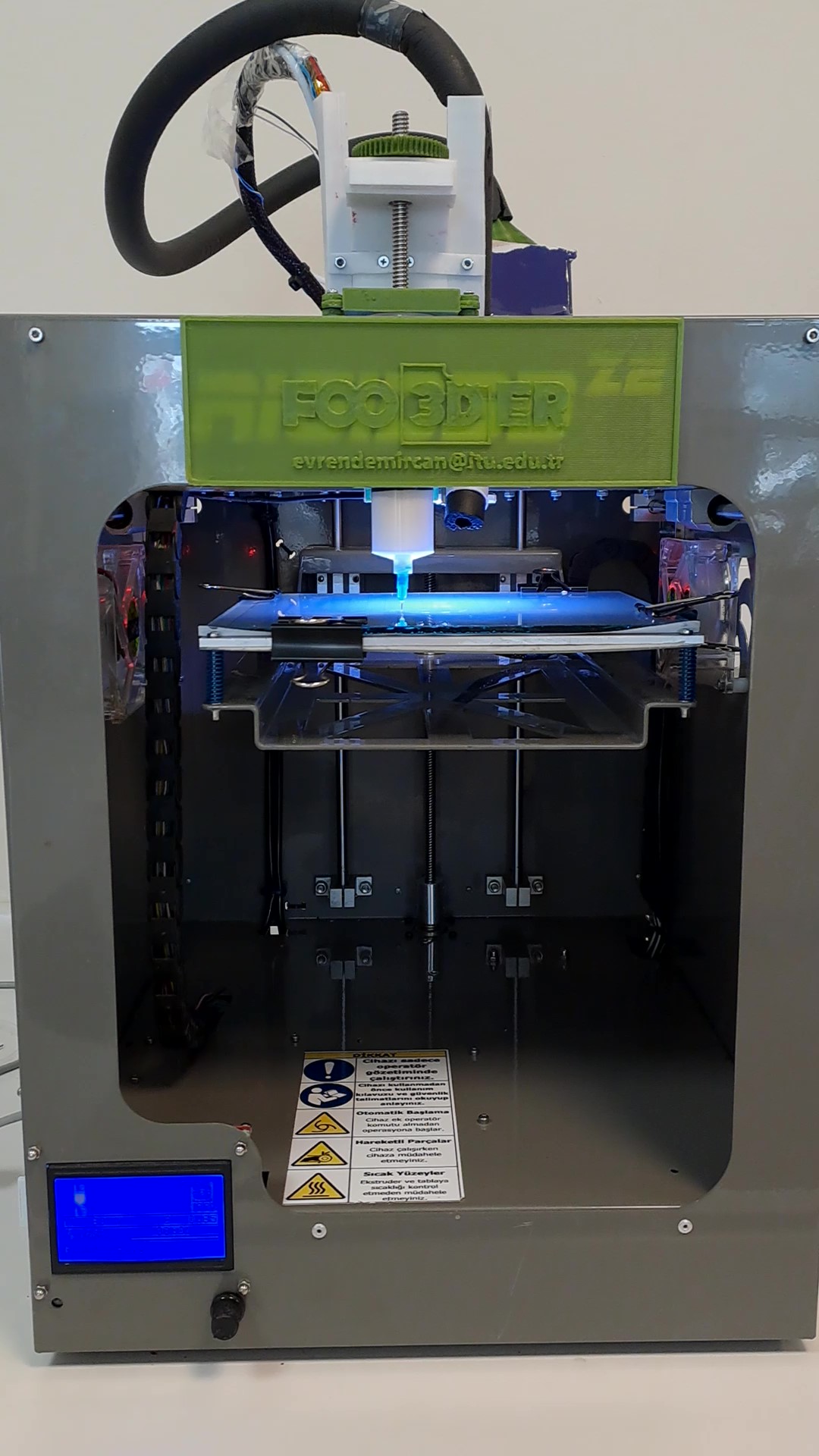


**Figure S2.** The 3D Printer on which the SPM mounted (top-left view).


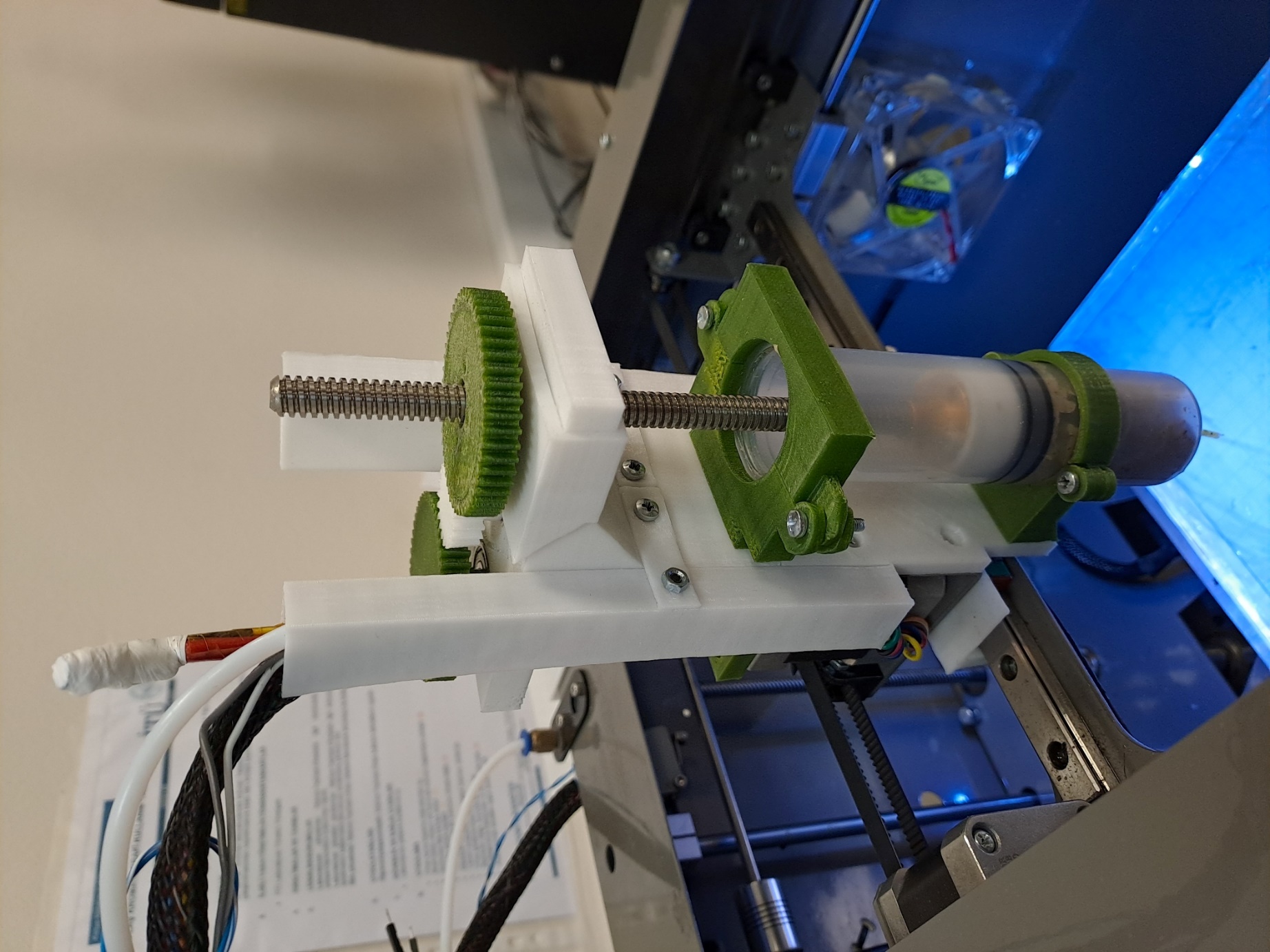


**Figure 3.** The 3D Printer on which the SPM mounted (top-right view).


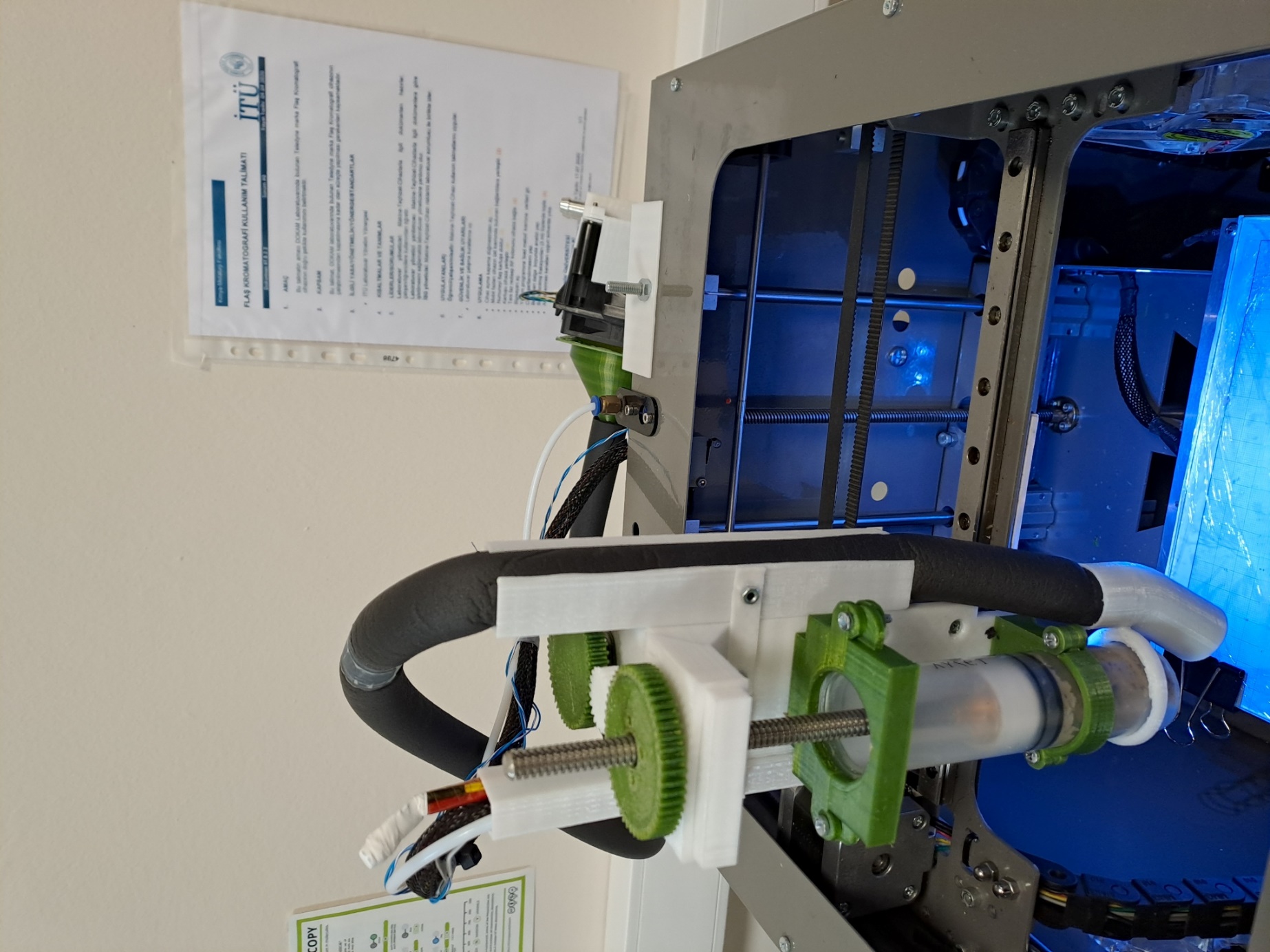

Supplement: Supplementary data 1 [file mmc1.docx]
